# Improving community readiness among Iranian local communities to prevent childhood obesity

**DOI:** 10.1186/s12889-023-15163-3

**Published:** 2023-02-15

**Authors:** Mahdieh Niknam, Nasrin Omidvar, Hassan Eini-Zinab, Naser Kalantari, Keyvan Olazadeh, Parisa Amiri

**Affiliations:** 1 Research Center for Social Determinants of Health, Research Institute for Endocrine Sciences, Shahid Beheshti University of Medical Sciences, Velenjak St., Shahid Chamran Highway, Tehran, Iran; 2 Department of Community Nutrition, National Nutrition and Food Technology Research Institute (NNFTRI), Faculty of Nutrition Sciences and Food Technology, Shahid Beheshti University of Medical Sciences, West Arghavan, St. Farahzadi Blvd., Shahrak Qods, Tehran, Iran

**Keywords:** Childhood obesity, Prevention program, Community readiness, Local communities, Intervention

## Abstract

**Background:**

Community Readiness Intervention for Tackling Childhood Obesity (CRITCO) is a theory-based intervention being developed to improve the readiness of an Iranian urban population to engage in childhood obesity prevention programs. This study aimed to explore changes in readiness of intervention and control local communities from diverse socio-economic areas of Tehran.

**Methods:**

This study was a seven-month quasi-experimental intervention implemented in four intervention communities and compared with four controls. Aligned strategies and action plans were developed around the six dimensions of community readiness. The Food and Nutrition Committee was established in each intervention community to make collaborative efforts among different sectors and assess the fidelity of the intervention. The pre-and post- readiness change was explored through interviews with 46 community key informants.

**Results:**

The total readiness of intervention sites increased by 0.48 units (*p* < 0.001) and shifted to the next higher level, from preplanning to the preparation stage. At the same time, the readiness of control communities decreased by 0.39 units (*p* < 0.001), although their readiness stage remained unchanged, reflecting the fourth stage. Also, a sex-dependent CR change was observed, such that the girls’ schools showed a more remarkable improvement in interventions and less decline in controls. The readiness stages of interventions significantly improved for four dimensions related to community efforts, knowledge of the efforts, knowledge of childhood obesity issue, and leadership. Furthermore, the readiness of control communities significantly decreased on three of six dimensions related to community effort, knowledge of efforts, and resources.

**Conclusions:**

The CRITCO successfully improved the readiness of intervention sites for addressing childhood obesity. It is hoped that the present study can be a spark for developing readiness-based childhood obesity prevention programs in Middle Eastern and other developing countries.

**Trial registration:**

The CRITCO intervention was registered at Iran Registry for Clinical Trials (http://irct.ir; IRCT20191006044997N1) on 11/11/2019.

**Supplementary Information:**

The online version contains supplementary material available at 10.1186/s12889-023-15163-3.

## Background

Childhood obesity is a public health problem with numerous adverse health and social consequences [1, 2]. Finding from a recent nationwide study revealed an upward trend of childhood obesity in Iran and showed that 20.8% and 21.1% of students, aged 7–18 years had excessive weight and abdominal obesity, respectively [3]. Community involvement has recently been emphasized to prevent childhood obesity [4]. In this regard, the community-based participatory research (CBPR) approach is proposed to establish and maintain partnerships between researchers, professionals, and the community to stimulate a sense of empowerment and ownership and improve the sustainability of health programs [5, 6]. Communities are at different levels of readiness to accept or support an issue. Thus a community-based intervention may fail if one community is not ready to recognize an issue as a problem [7].

In designing community-based programs, measuring community readiness (CR) identifies how a community can address an issue of interest [8, 9]. A readiness assessment helps guide goals and prevention strategies to motivate and mobilize the community to a higher level of readiness. Accordingly, exploring changes in CR provides a framework to inform planners about the effectiveness of an intervention [7–9]. Several conceptions of readiness and tools are developed to determine the readiness stage for a specific issue or problem [9–13]. The community readiness model (CRM) is a frequently used conceptual framework established in the Tri-Ethnic Center for Prevention Research at Colorado State University [9]. The CRM is closely aligned with the CBPR model due to its requirement to involve community members in identifying community needs and insights to assess readiness and develop appropriate and sustainable strategies [10, 12]. According to the escalating trend of childhood obesity in Iran, a national primary healthcare-based program entitled IRAN-Ending Childhood Obesity (IRAN-ECHO) in the framework of the WHO-ECHO program was initiated in 2015 [14]. However, no understanding of CR exists in the Iranian population in national or local programs. Considering the multiculturality of Iranian society, the unequal distribution of resources, and socio-cultural misconceptions about childhood obesity, CR-specific strategies are prerequisites for national and regional programs. Thus, for the first time, the Community Readiness Intervention for Tackling Childhood Obesity (CRITCO) was designed and conducted in Tehran, the capital of Iran, to assess and improve the readiness of targeted local communities to engage in childhood obesity prevention programs. The CRITCO study hypothesis is that implementing a tailored intervention by improving the readiness of the target communities can finally lead to increase the effectiveness of such programs. This paper aimed to examine the extent to which a tailored intervention can improve the readiness of Iranian local communities recruited from diverse socioeconomic districts of Tehran to engage in obesity prevention programs.

## Methods

### Conceptual framework

The study was developed based on CRM. The CRM was built based on the Transtheoretical Model of Stages of Change and theories on Community Development and Social Action. The CRM followed extensive research and testing, establishing reliability within multiple communities of differing issues [9]. As a mixed-method approach, CRM incorporates a qualitative component and numerical scoring [15]. The model has comprised six dimensions, including 1) existing community efforts, 2) community knowledge of efforts, 3) leadership, 4) community climate, 5) community knowledge of the issue, and 6) available resources. Through in-depth interviews, each dimension was scored by exploring the community’s key informants’ (KIs) opinions. The final CR score derives from the average score of the six dimensions and rounds down to a number corresponding to one of the nine stages of readiness (Table 1) [7, 9].

**Table 1:** Description of the nine stages of community readiness with corresponding scores

**Stage (Readiness Score)**	**Description**
1. No awareness (1–1.99)	The issue is normative and accepted and not generally recognized as a problem
2. Denial (2–2.99)	A few community members recognize that the issue is a concern; the community and leadership do not support dealing with the problem
3. Vague awareness (3–3.99)	Community members and leaders believe that the issue may be a concern, but there is no immediate motivation to address the issue
4. Preplanning (4–4.99)	Community members and leaders acknowledge that the issue is a concern; the efforts are not focused or detailed
5. Preparation (5–5.99)	Most community members have basic knowledge of the issue and are concerned about the issue; leaders are planning; some resources have been identified, and community members and leaders are actively working to secure resources
6. Initiation (6–6.99)	Leaders play a key role in planning efforts to address the problem; enough information is available; resources have been allocated to support efforts
7. Stabilization (7–7.99)	Most community residents have more than basic knowledge of existing efforts and issues; leaders actively improve the efforts
8. Confirmation/ Expansion (8–8.99)	Efforts are in place; community members strongly support efforts; leaders play a key role in expanding and improving efforts
9. Community ownership (9–9.99)	Most community members know local efforts and issues; leaders continually review evaluation results and secure resources

### Overview of the CRITCO study

The CRITCO was a multilevel, multisector, theory-based study with an advisory committee comprised of two community nutritionists, a sociologist, a health education and promotion specialist, and a pediatrician. The overall aim of the study was to improve the readiness of targeted local communities of diverse socioeconomic districts of Tehran for engaging in childhood obesity prevention programs in primary school children grade fourth to sixth (10–12 years of age). Accordingly, the specific objectives were developed around the six dimensions of CRM to promote community knowledge about the existing preventive efforts and childhood obesity issues, leaders’ support, community partnership, and resource allocation to address childhood obesity. The CRITCO study was launched and implemented over two years (August 2018 to August 2020) in five distinct phases, where each phase developed based on the findings of the previous phases:

The first two phases developed a validated socio-culturally adapted Persian version of the community readiness tool (CRT). Subsequently, a cross-sectional study determined the readiness of 12 local communities from two diverse socioeconomic districts of Tehran city (districts 2 and 16). The fourth phase developed an intervention package based on the readiness level data and environmental scan. In the fifth phase, the effectiveness of an evidence-based intervention was determined by exploring changes in the readiness of intervention and non-intervention local communities.

### Recruitment to the study

The CRITCO intervention (http://irct.ir; IRCT20191006044997N1) was a prospectively registered controlled quasi-experimental trial carried out in eight local communities from two diverse socioeconomic districts of Tehran, Iran. The first local community was enrolled on November 16, 2019, and the last local community was enrolled on November 21, 2019. The study was initiated and conducted between November 2019 and May 2020. Contrary to what has been mentioned in our registered protocol, the duration of intervention was extended from six to seven months. Eight of the 12 involved local communities (4 interventions; 4 controls) were selected to participate in the intervention program. Thus, two local communities from each district with a population of 2675 late primary school children were selected according to their willingness to engage in the intervention and site-specific readiness scores. Their willingness was verified through interviews with each local community leader. Subsequently, according to the location and readiness level data, the control group included four local communities with 2560 late primary students selected from the other involved communities. Each “local community” involved four sectors; an elementary school (public boy’s or girl’s school), the students’ families, a public health care center, and a municipal community center in each school neighborhood. Schools were the primary setting for implementing the interventions. The intervention targeted changes at four levels or audiences; students in grades 4 to 6, school personnel, parent-families, principals and/or nutritionists.

### Intervention

At the beginning of the intervention, community engagement and support seeking were conducted through official writing from the General Office of Education to the school principals and also in-person meetings with principals and nutritionists of each local community. Furthermore, attendance meetings were held with the school Parents-Teachers Association members and students’ Health Ambassadors. Building relationships and integrated efforts among different sectors and levels was the core approach of the intervention. In this regard, six to eight representatives were chosen purposively based on community engagement and interest in health issues to establish a Food and Nutrition Committee (FNC) (Additional file 1). The FNCs members, as integrators, bridged among different sectors and focused on all scheduled activities to provide essential support to make a collaborative effort and negotiation.

The action plan was developed as a living document around the six dimensions of the CRM based on five core strategies, including education, encouragement, creating supportive environments, informing, and sensitizing (Additional file 2). Interventions in schools focused on improving the readiness of three target audiences: students, school personnel, and parents-families. The main components of these interventions included: 1) the physical activity and healthy eating promotion programs (School Breakfast/Snack program, Food Festivals, Dynamic Yard program, specific programs for particular events, and in-school competitions); 2) the educational programs (individual- or group-based educations, and creating consistent messages through posters or educational materials); 3) development and/or improvement of community partnership (establishment of small informing groups among parents and students, creating school health channels in social media, merging several programs to direct the school and parental time). The family intervention targeted changes in parents and students. Its main intervention components included: 1) developing a variety of healthy eating and physical activity challenges in social media; 2) developing virtual educational programs on childhood obesity and healthy behaviors; 3) organizing outdoor sports programs and health stations. The intervention in public health care and municipal community centers targeted changes in students, parents-families, principals and/or nutritionists. The main intervention components included: 1) the development of several educational programs, 2) improvement of the school buffets’ quality (providing or revising the allowed food lists of school buffets, appointing a task force for regular evaluation of the school buffets); 3) improvement of information/statistics sharing and bidirectional referral system between schools and nutritionists.

Fidelity assessments were conducted by scanning the environment and filling out a checklist by a member of the advisory committee and FNCs. In this regard, the FNCs monitored and recorded all qualitative and quantitative aspects of activities using a data-collection form that involved the information comprising date, scale, duration, frequency, adherence, community partnership, executive director, and resources of actions (Additional file 3). Also, environmental scans were conducted based on direct observation of schools, social settings, and neighborhood environments in their surroundings. The process evaluation data were compiled and completed into one data file and presented to the advisory committee and FNC members at the monthly meeting.

### Study participants and data collection

The data collection was done via conducting structured interviews with KIs pre-and post-intervention. Despite in-person interviews at baseline, follow-up interviews were conducted by phone due to the COVID-19 outbreak. A trained community nutritionist of the project advisory committee conducted all interviews. Based on the CRM protocol, key informants were identified using purposeful and snowball sampling techniques among school personnel, parents, principals and/or nutritionists of public health care and municipal community centers. To provide confident answers and insights, key respondents had at least one year of experience working in each local community and were involved in children’s health and wellbeing. Forty-six interviews were conducted at baseline (22 interventions; 24 controls). In this regard, all baseline participants were approached for follow-up interviews. Each interview lasted an average of 40 min and written informed consent was obtained before each interview (Additional file 4). All interviews were audio-recorded and transcribed verbatim. In addition, the data derived from the environmental scan and monitoring were used as supplementary information alongside the interview data.

### Measurement tool and scoring

A validated Persian version of the CRT was used to assess CR changes [16]. The validated CRT included thirty-seven questions on six CRM dimensions. Two reviewers who were members of the research reviewed all transcripts and followed the standard anchored scoring protocol of the Tri-Ethnic Center’s handbook. In this regard, the qualitative content analysis of interview data was conducted to determine the scores for each dimension based on the assigned criteria [9]. The qualitative data analysis was managed by MAXQDA _2010_. Subsequently, mean and standard deviation (SD) were calculated for each dimension, and the average of the six dimensions’ consensus scores gave the final readiness score, which indicated the corresponding readiness stage of each intervention and control group (possible range = 1–9). Reviewers scored interviews after all interviews were completed for each community.

### Statistical analysis

The inter-rater reliability among scorers at baseline and follow-up was assessed based on Kendall’s coefficient of concordance. For assessing changes in overall CR and CR dimension scores from baseline to post-intervention, the normality of data was checked using the Kolmogorov–Smirnov test. Changes in overall CR and CR dimension scores between baseline and follow-up were assessed using Repeated Measure Analysis. In this regard, the Repeated Measure Analysis was used to test whether the main effects of the local community baseline CR or intervention type significantly explained the variance in changes across time. In addition, the independent t-test was used to explore the sex-specific differences in overall readiness scores of intervention and control sites. Results were determined statistically significant at *p* < 0.05. Statistical analyses were conducted by SPSS version 20.

**Fig. 1:**
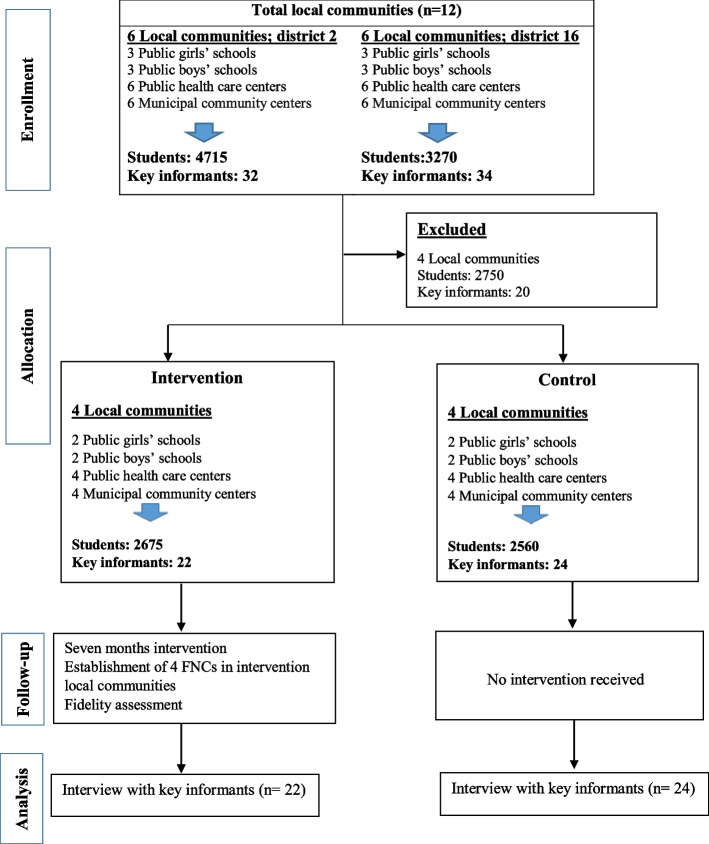
Study flow of the CRITCO intervention

A summary of the study flow is presented as a diagram in Fig. 1.

## Results

All baseline key respondents participated in follow-up interviews, and 46 interviews were conducted in intervention and control groups. The inter-rater reliability among scorers was moderate to high (baseline and follow-up scores by dimensions: 1) Existing community efforts = 0.74, 0.78; 2) Community knowledge of efforts = 0.71, 0.78; 3) Leadership = 0.81, 0.85; 4) Community climate = 0.84, 0.87; 5) Community knowledge of the issue = 0.69, 0.73; 6) Available resources = 0.72; 0.79; all *p*-values were < 0.001).

Table 2 presents the categorical socio-demographic characteristics of the interviewed key informants by intervention and control sites. The majority of the KIs were female (96%) and married (91%), and almost half of them were school personnel. Participants in the control group were relatively older, and all had college education compared to 86% in the intervention group.

**Table 2:** Socio-demographic characteristics of the interviewed key informants across intervention and control sites (*n* = 46)

	**Control n (%)**	**Intervention n (%)**
**Overall**	24 (100)	22 (100)
**Sex**		
Female	23 (96)	21 (95.5)
Male	1 (4)	1 (4.5)
**School Staff**		
Principals	4 (16)	2 (9)
Teachers	7 (29.3)	9 (40.5)
**Parents**	5 (21)	3 (13.5)
**Principals from the Municipal community center**	4 (16.8)	4 (18)
**Nutritionists from the Public health care center**	4 (16.8)	4 (18)
**Age (**years**)**		
20–29	0 (0)	1 (4.5)
30–39	4 (16.8)	9 (40.5)
40–49	15 (62.5)	12 (54)
50–59	5 (21)	0 (0)
**Level of education**		
High school diploma	0 (0)	3 (13.5)
BSc	21 (87.5)	17 (77)
MSc	3 (12.5)	2 (9)
**Marital status**		
Single	2 (8.4)	2 (9)
Married	22 (91.6)	20 (91)
**Length of association with the local community (**years**)**		
1–3	12 (48)	9 (40.5)
3–6	8 (35)	9 (40.5)
6–9	3 (12.5)	1 (4.5)
> 9	1 (4.5)	3 (13.5)

The fidelity assessment results revealed that nearly 81% of scheduled activities had been performed with medium quality (score: 3.5 from 5). The results showed that, compared with the boys’ schools, girls’ schools had more adherence (85% vs. 78%) and higher quality of performance (score: 3.8 vs. 3.2) (Additional file 5). The data on fidelity and quality implementation by CRM dimensions can be found in Additional file 2.

### The community readiness results

Baseline and follow-up readiness scores of intervention and control local communities are presented in Table 3 (to better disaggregate local communities, each local community is presented based on the district and sex of its involved school). Data analysis showed that from the key informants’ views, the overall readiness score of intervention sites increased by 0.48 units (*p* < 0.001), such that the stage of readiness shifted to the next higher level, from the fourth to the fifth stage. At the same time, the readiness of control sites decreased by 0.39 units (*p* < 0.001), although the overall stage of readiness remained unchanged, reflecting the fourth stage (Table 4). Also, a sex-dependent improvement was observed in readiness scores of interventions such that girls’ schools showed a more remarkable improvement compared to boys’ schools (5.32 ± 0.12 vs. 4.80 ± 0.14, *p* < 0.001). Furthermore, the readiness of control sites was significantly decreased in boys’ compared to girls’ schools (4.49 ± 0.36 vs. 4.10 ± 0.26, *p* = 0.007).

**Table 3:** Overall readiness scores of interventions and controls at baseline and follow-up by sex and ^a^SES

**Local communities**	**Overall readiness scores**		**Change**	
	Baseline	Follow-up		
**Interventions**				
Girl’s school, ^c^High SES	^b^4.80 ± 0.13	5.28 ± 0.16	+ 0.48	
Boy’s school, High SES	4.28 ± 0.18	4.81 ± 0.06	+ 0.53	
Girl’s school, ^d^Low SES	4.83 ± 0.15	5.35 ± 0.07	+ 0.52	
Boy’s school, Low SES	4.42 ± 0.18	4.79 ± 0.21	+ 0.37	
**Controls**				
Girl’s school, High SES	5.00 ± 0.13	4.82 ± 0.10	-0.18	
Boy’s school, High SES	5.02 ± 0.10	4.27 ± 0.20	-0.93	
Girl’s school, Low SES	4.59 ± 0.19	4.16 ± 0.11	-0.43	
Boy’s school, Low SES	4.10 ± 0.13	3.93 ± 0.22	-0.17	

^a^Socio-economic status

^b^Mean ± SD

^c^District 2

^d^District 16

**Table 4:** Baseline and follow-up readiness differences of interventions and controls by total and dimensions

**CRM dimensions**	**Interventions**			**Controls**		
	Baseline/Follow-up	Change	^b^*P*-value	Baseline/Follow-up	Change	*P*-value
**Existing community efforts**	^a^4.85 ± 0.49/ 5.77 ± 0.47	+ 0.92	** < 0.001**	4.93 ± 0.62/ 4.06 ± 0.57	- 0.87	** < 0.001**
**Community knowledge of efforts**	4.74 ± 0.51/ 4.95 ± 0.51	+ 0.21	**0.02**	5.05 ± 0.61/ 4.57 ± 0.48	- 0.48	** < 0.001**
**Leadership**	4.46 ± 0.42/ 5.07 ± 0.30	+ 0.61	** < 0.001**	4.45 ± 0.47/ 4.28 ± 0.39	- 0.17	0.06
**Community climate**	4.41 ± 0.33/ 4.58 ± 0.30	+ 0.17	0.06	4.28 ± 0.39/ 4.23 ± 0.33	- 0.05	0.56
**Community knowledge of issue**	4.73 ± 0.46/ 5.56 ± 0.58	+ 0.83	** < 0.001**	4.78 ± 0.66/ 4.59 ± 0.57	- 0.19	0.18
**Resources**	4.29 ± 0.42/ 4.43 ± 0.34	+ 0.14	0.09	4.57 ± 0.55/ 4.18 ± 0.48	- 0.39	** < 0.001**
**Total CR**	4.58 ± 0.29/ 5.06 ± 0.29	+ 0.48	** < 0.001**	4.68 ± 0.40/ 4.29 ± 0.36	- 0.39	** < 0.001**

^a^Mean ± SD

^b^Repeated measure analysis

### The community readiness by CRM dimensions: qualitative and quantitative data

**Fig. 2:**
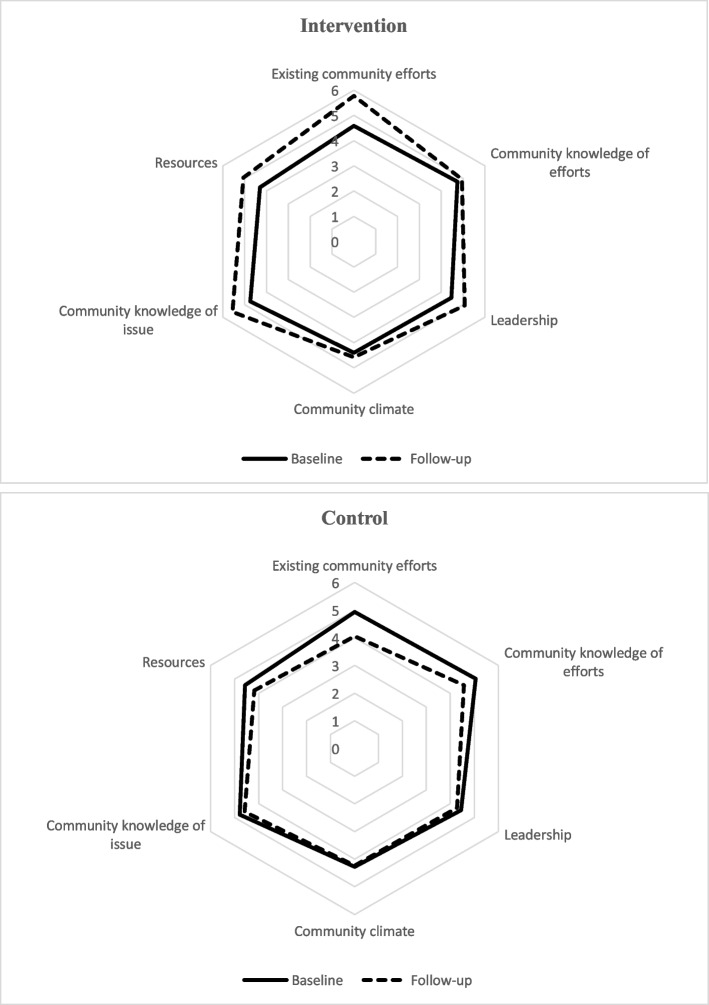
Changes in the readiness of the intervention and control groups by CRM dimensions over the intervention period

Baseline and follow-up readiness scores of intervention and control communities by CRM dimensions are presented in Table 4. Data analysis revealed that the readiness scores of the intervention group significantly improved for four out of six CRM dimensions, including “existing community efforts”, “community knowledge of efforts”, “leadership”, and “community knowledge of issue”. The analysis for the control group also illustrated that readiness scores significantly decreased for three of six CRM dimensions, including “existing community efforts”, “community knowledge of efforts”, and “resources”. Changes in readiness of control and intervention communities by CRM dimensions over the intervention period are presented in Fig. 2.

The following focuses on the dimension-specific scores and interpreting scores using the content analysis of the KIs’ interviews:

- *Existing Community efforts* increased by 0.92 units (*p* < 0.001) for the intervention group, while the scores of the control decreased significantly by 0.87 units (*p* < 0.001). Many respondents believed that the establishment of the FNC in each intervention site and their efforts for supporting and coordinating activities, engaging community members, and sharing the relevant data had a critical role in promoting efforts and programs: “*The FNC shared the data with the leaders and parents and evaluated all activities”, “Irregular and temporary activities were managed effectively by FNC…”.* In contrast, almost all key respondents in the control communities pointed to the declining trend of programs and efforts made in that academic year compared to previous years. The main factors that made this difference were the lack of school time due to consecutive and unplanned holidays and the COVID-19 outbreak: “M*ost of the healthy food programs were canceled due to concern about Corona outbreak…”.* Also, parents’ poor participation due to being too busy was the main barrier to expanding and implementing the prevention efforts in control communities. While this barrier decreased to an acceptable level in the intervention groups by integrating several programs into one to manage school and parental time: *“…The parents’ attendance at the school group meetings and educational programs was managed by merging several programs”.*
- *Community Knowledge of efforts* of intervention communities significantly improved by 0.21 units (*p* = 0.02), while controls decreased by 0.48 units (*p* < 0.001). Although some ways existed previously to build awareness among community members about the current prevention efforts, several initiatives suggested by FNCs enhanced the available routes and improved the knowledge of intervention communities about existing programs and nutrition-related activities. In this regard, the establishment of a separate school health channel in social media, and small informing groups improved community-wide communication and data sharing: *“Through the last academic year, an active health channel provided essential information about health programs”, “Establishment of small informing groups among interested and supportive parents was an initiative approach to build awareness, and improve their participation…”.* In comparison, challenges faced by parents, teachers, and school principals for attending school group meetings were the most common problems declared by the key respondents for exchanging related information in the control communities: *“Lack of school time is a common problem…”, “Poor attendance of parents in school group meetings is our permanent challenge”.* Furthermore, the lack of community leaders committed to distributing detailed and updated nutrition- and health-related information was frequently stated as a barrier by key informants of the control sites: *“The principal posts the news and photos on the school channel, but not in detail and not in an updated fashion”.*
- *Leadership* increased significantly by 0.61 units (*p* < 0.001), shifting the readiness stage of interventions by one level from preplanning to preparation. In contrast, the scores of control communities decreased by 0.17 (*p* = 0.06) and remained at the same level. The key respondents frequently pointed to several efforts developed or facilitated by local community leaders during the intervention period. In this regard, several efforts, including the “Dynamic Yard Program”, School Breakfast Program, Food Festivals, creating a health channel on social media, holding group discussions with parents, and appointing a task force to check the school buffet were conducted or facilitated by leaders: *“The school principal talked to teachers and parents about the School Breakfast Program… She supervised the implementation of Food Festival and Dynamic Yard Program”, “Nutritionist of the public health center aligned with the principal of the municipal community center developed monthly educational programs for local community members”.* From the respondents’ view, the establishment of FNCs with the attendance of some local leaders resulted in their active support and diverted their attention to childhood obesity issue; *“The membership of school principals in FNC resulted in their involvement in the programs and coordinate activities”.* On the other hand, most comments from control communities indicated the passive support of the leaders: *“Childhood obesity is a matter of concern for school principal but is not a priority, and there is not enough motivation to do something …”*.
- *Community climate* improved by 0.17 units (*p* = 0. 06) for the intervention communities, while the scores of controls decreased by 0.05 units (*p* = 0.56). After the intervention, despite most of the key respondents believed that the intervention several efforts (establishment of small informing groups, holding group or educational programs, distributing health messages around the communities, managing the school, and parental time) were conducted to improve the community members’ partnership, they described several challenges that might contribute to the lack of change in readiness level across the community climate dimension. In this regard, the lack of parental engagement due to perceived misconceptions about childhood obesity was cited as the main barrier to community involvement: *“… Some parents like their kids to be obese and believe that bigger child can better protect themselves against physical damage”.* Furthermore, other obstacles including divorce, unemployment and poverty, resistance to learning, lack of security, budget, time, and facilities were mentioned frequently by respondents: *“Divorce is a common problem in our school…”, “Economic problems and lack of security are important barriers for children’s activity”, “the community resistance against training is a serious challenge in our community partnership”.*
- *Community knowledge of the issue* increased significantly by 0.83 units (*p* < 0.001) and improved by one level from preplanning to the preparation stage. The readiness score of control communities decreased by 0.19 (*p* = 0.18) and remained at the same level of readiness.
- Over the intervention period, various activities (e.g., the establishment of informing groups and school health channels, educational sessions, group discussions, and distributing health messages) were conducted to improve the knowledge of community members about childhood obesity issue: *“Initiating the school health channel was an effective way for sharing the information and improving knowledge of community members”.* Key participants believed that sensitizing the community members and creating supportive environments improved the knowledge of local communities about the causes, consequences, signs, and symptoms of childhood obesity: *“Presenting the information helped the leaders and community members see the problem at their local level and increased their knowledge about the consequences of obesity in children”, “Distributing posters, messages, and educational materials increased awareness about childhood obesity.”*
- *Resources related to obesity prevention* increased by 0.14 units (*p* = 0.09) for intervention communities, and the scores of controls decreased by 0.39 units (*p* < 0.001). Hence, the readiness stage of both communities remained unchanged and reflected the preplanning stage. The lack of space, time, human and financial resources were mentioned as the main limitations in all local communities (control and intervention). *“No funds are allocated to healthy eating programs, and families fund these programs”, “Only a small budget allocated for buying sports equipment …”, “Limited time is a common problem…”, “The schoolyard is very small, … We have no indoor sports hall”.* In the intervention communities, despite the efforts being to some extent successful in developing human and time resources, they were not very fruitful in attracting financial resources and developing space facilities. “*The FNCs efforts could attract professional stakeholders and volunteers”*. On the other hand, most of the respondents in the control communities mentioned a greater lack of financial, time, and human resources compared to the previous similar academic year; *“In the last academic year, due to many unplanned holidays, we faced more time constraints”, “…Because of the high cost of food, we could not hold the healthy food festival this year…”,” Activities related to nutrition were less supported by the school principal due to his busy schedule”.*

## Discussion

The present study revealed that the readiness of intervention communities increased between baseline and follow-up and received to the preparation stage. Furthermore, the dimension-specific readiness stages of interventions significantly improved for four CRM dimensions related to community efforts and programs, community knowledge about the existing efforts and childhood obesity issue, and leadership. At the same time, the readiness score of the control communities significantly decreased, although their overall stage of readiness remained unchanged, corresponding to the preplanning stage. Furthermore, the dimension-specific readiness scores of control communities significantly decreased on three of six dimensions related to the community effort, knowledge of efforts, and resources. Also, a sex-dependent increasing and declining CR change was observed in intervention and control communities, such that the girls’ schools showed a more remarkable improvement in interventions and less decline in controls.

Although most studies have measured CR to determine the current state of the community for the prevention of childhood obesity [17–22], available evidence regarding CR levels pre-and post-intervention(s) is rare and limited to high-income countries [23–27]. Furthermore, no studies launched an intervention considering the sex of children and the SES of target communities. SaludABLEOmaha’s study is an example that used CRM to improve the readiness of American residents in the Midwestern Latino community to address adolescent obesity through two years of intervention [24]. Ready for Recess’ study determined the effectiveness of a one-year intervention in changes in school readiness on the amount of physical activity during recess and school days [23]. Furthermore, “It is Your Move” was a 3-year controlled obesity prevention project in Australian secondary schools using the CRM and readiness data as a diagnostic measure of capacity to promote healthy eating and physical activity [26]. The above studies showed that assessing the CR changes is a precursor to the effective application of evidence-based practices for preventing childhood obesity.

The CRITCO study, as the first multilevel, multisector readiness-based prevention effort among developing countries, resulted in the readiness increment of intervention communities by 0.48, suggesting that the intervention successfully improved CR by one level, from the preplanning to the preparation stage. This result is consistent with findings from a previous systematic review that suggested an increase between 0.5 and 1 in CR levels per year intervention [28]. This CR improvement can be attributed to four main factors considered in developing and implementing the intervention: First, due to the complexity and interconnectedness of etiologic factors of childhood obesity [29], this study used an integrated approach across multiple sectors and stakeholders for improving the readiness of target local communities. Second, as integration requires partnership and collaboration [24], essential support for building relationships and participatory efforts among targeting audiences was possible by FNCs. Third, the strategies were developed congruently with the readiness and socio-cultural characteristics of the targeted local communities. Fourth, the action plan was used as a living document that evolved throughout the project, and intervention structures were tailored to each local community’s needs, resources, SES, and interests.

The present study showed that the readiness stage of intervention sites increased significantly to a higher level for the dimensions related to community efforts, knowledge, and leadership. Although similar to the other readiness-based obesity prevention efforts, most conducted efforts focused on promoting healthy eating and physical activity [23, 30]; the current study went beyond this, and by creating cross-sectoral collaboration and investment in people, improved the level of readiness for the community effort dimension. In addition, our findings are consistent with the results from other studies that showed due to a strong direct relationship between building awareness and leadership, small changes in knowledge could facilitate more extensive changes in community attitude and leadership [26, 31]. Improving the readiness of intervention sites on the leadership can be attributed to the establishment of FNC, which some local leaders attended, and frequent individual meetings with them were made possible. In addition, improving the readiness of intervention communities for two dimensions related to knowledge and awareness (community knowledge of the effort and issue) can be justified by establishing small informing groups among local community members, individual- or group-based education, creating the school health channel in social media, distribution of health messages, posters and educational materials around the intervention communities.

In the present study, although the readiness stage of the control group remained unchanged, their total and dimension-specific readiness scores of the three dimensions decreased significantly between baseline and follow-up. This finding is in line with the data from a controlled obesity prevention study that showed the readiness of most comparison communities decreased between pre-and post-intervention [26]. Communities are fluid and constantly changing, adapting, and growing; hence they are ready for different things at different times [7]. This highlights the need to do constant activities to increase community awareness and gain the support of the leaders and community members to recognize an issue as a priority [20, 32]. Hence the observed decrease in dimensions related to the community effort, knowledge of efforts, and resources can be explained by the factors repeatedly mentioned by the key informants, including poor community participation, limited time, consecutive and unplanned holidays, weather conditions, and the COVID-19 outbreak.

In the present study, a sex-dependent relationship was observed in the CR change, such that the readiness of girls’ schools compared to boys’ schools was accompanied by a greater increase and a smaller decrease in interventions and controls. Due to the various sex disparities in weight-related cultural beliefs and perceptions, it is not far from expected that the leaders and community members were more concerned and motivated to be involved in obesity prevention efforts for girls rather than boys [33, 34]. In addition, the heterogeneity of each intervention local community in adherence, scale, and frequency of activities, and more compliance and higher performance quality in girls’ schools than in boys’ schools could explain this finding.

### Strengths and limitations

The present study is the first attempt to apply the CRM toolkit in a Middle-Eastern country. In addition, it is the first study that used the readiness level data to align the activities and explore changes in a childhood obesity prevention project in developing countries. However, the study has certain limitations: Although a cluster randomized controlled trial design would have been technically ideal, a quasi-experimental design was used because of funding restrictions. Also, the purposive sampling of KIs could raise the possibility of selection bias; however, it was minimized to some extent by recruiting a wide range of informants. The generalizability of the results is limited to communities with similar cultural and socioeconomic characteristics.

## Conclusion

The present study results showed that CRITCO successfully improved the readiness stage of intervention communities for addressing childhood obesity. The participatory efforts were made possible by engaging the community leaders, establishing FNCs, and making participatory efforts. It is hoped that the present study can be a spark for developing readiness-based childhood obesity prevention programs in Middle Eastern and other developing countries.

## Supplementary Information

**Additional file 1. **Food and Nutrition Committee (FNC) members.

**Additional file 2. **Intervention action plan around the six dimensions of the community readiness model.

**Additional file 3. **Data collecting form for assessing the quantitative and qualitative aspects of activities.

**Additional file 4. **Interview consent form.

**Additional file 5. **The results of fidelity assessment by sex and ^a^SES.

## Data Availability

The datasets used and/or analyzed during the current study are available from the corresponding author on reasonable request.

## Abbreviations

CRITCO: Community Readiness Intervention for Tackling Childhood Obesity
CBPR: Community-based participatory research
CR: Community readiness
CRM: Community readiness model
CRT: Community readiness tool
FNC: Food and Nutrition Committee
