# Supplementary material for: Improving community readiness among Iranian local communities to prevent childhood obesity
Source: BMC Public Health. 2023 Feb 15;23:344. doi: 10.1186/s12889-023-15163-3 (PMC9931445; doi:10.1186/s12889-023-15163-3)
Supplement: Supplementary file 1 — Additional file 1. Food and Nutrition Committee (FNC) members. [file 12889_2023_15163_MOESM1_ESM.docx]

Additional file **1:** Food and Nutrition Committee (FNC) members

| **District 16** | | **District 2** | | **FNC members** |
| --- | --- | --- | --- | --- |
| 1 | 1 | 1 | 1 | School principals |
| 1 | 1 | 1 | 1 | School health & education consultant |
| 1 | 1 | 1 | 1 | Physical education teacher |
| 2 | 1 | 2 | 3 | Parent |
| 1 | 1 | 1 | 1 | Nutritionist of public health center |
| 1 | 1 | 1 | 1 | Principal of Municipal community center |
| **7** | **6** | **7** | **8** | **Total** |
