# Supplementary material for: Improving community readiness among Iranian local communities to prevent childhood obesity
Source: BMC Public Health. 2023 Feb 15;23:344. doi: 10.1186/s12889-023-15163-3 (PMC9931445; doi:10.1186/s12889-023-15163-3)
Supplement: Supplementary file 2 — Additional file 2. Intervention action plan around the six dimensions of the community readiness model. [file 12889_2023_15163_MOESM2_ESM.docx]

Additional file **2:** Intervention action plan around the six dimensions of the community readiness model

| **No.** | **Expected activities** | **Executive responsible in FNC*** | **Target audiences** | **Target strategies** | **Setting** | **Frequency/ Time** |
| --- | --- | --- | --- | --- | --- | --- |
| **1. Promoting childhood obesity prevention efforts in target local communities** | | | | | | |
| 1 | Develop a School Breakfast Program/ healthy school snack | Physical education teacher, School consultant in health & education, Nutritionist of community health center | Students, Parents, School personnel | Education, Creating supportive environments | School | weekly |
| 2 | Develop a variety of challenges focusing on healthy eating and physical activity | Physical education teacher, School consultant in Health & education, Parents, Nutritionist of community health center | Students, Parents | Encouragement, Informing, Sensitizing | School | monthly |
| 3 | Development painting or essay competitions with the topic of healthy foods and physical activity | Physical education teacher, School principal, Parents | Students, Parents, School personnel | Education, Creating supportive environments, Encouragement | School | 3 competitions |
| 4 | Development of in-school sports competitions | Physical education teacher, Parents | Students | Education, Creating supportive environments | School | 2 competitions |
| 5 | Development of a Dynamic Yard Program | Physical education teacher, School principal | Students | Creating supportive environments | School | From the beginning of the intervention |
| 6 | Allowance of to enter sports equipment to schools | Physical education teacher, School consultant in health & education, | Students | Creating supportive environments | School | From the beginning of the intervention |
| 7 | Development of happy and educational programs on particular days | Physical education teacher, School consultant in health & education, Nutritionist of community health center | Students, School personnel | Education, Encouragement | School | 3 programs |
| 8 | Holding Health stations on special days | School consultant in health & education, Parents | Students | Education, Creating supportive environments | School | 2 times |
| 9 | Holding group discussions with teachers with the participation of the community health center | School principal, School consultant in health & education, Nutritionist of community health center | Teachers | Education, Sensitizing | School | 2 times |
| 10 | Holding face-to-face educational sessions with the topic of healthy foods and physical activity for students | School consultant in health & education, Nutritionist of community health center | Students | Education, Sensitizing | School | 2 times |
| 11 | Providing educational books/booklet for the library of school and classroom | School consultant in Health & education, Parents, Nutritionist of community health center | Students, Teachers | Creating supportive environments | School | From the beginning of the intervention |
| 12 | Providing pamphlets, essays, and educational content in schools’ and municipal community centers’ channels in Telegram or WhatsApp | Principal of municipal community center, School consultant in health & education | Students, Parents, School personnel | Education, Creating supportive environments, Sensitizing | -- | From the beginning of the intervention |
| 13 | Holding educational program for parents in partnership with community health center | School principal, School consultant in health & education, Nutritionist of community health center | Parents | Education, Sensitizing | School | 2 times |
| 14 | Holding Food Festivals/ healthy school snacks in partnership with parents in schools | School principal, School consultant in health & education, Parents | Students, School personnel, Parents | Creating supportive environments | School | 2 times |
| 15 | Providing pamphlets, essays, and educational content by student health ambassadors | School consultant in health & education | Students, School personnel | Education, Creating supportive environments, Encouragement | School | From the beginning of the intervention |
| 16 | The quality improvement of schools’ buffet | School principal, School consultant in health & education, Parents | Students, School personnel | Creating supportive environments | School | From the beginning of the intervention |
| 17 | Organize outdoor sports for parents | Principal of municipal community center | Parents | Creating supportive environments, Encouragement | Local parks | 2 times |
| 18 | Holding face-to-face educational sessions for parents | Principal of municipal community center, Nutritionist of community health center | Parents | Education, Sensitizing | Municipal community center | Monthly |
| 19 | Holding Food Festivals/ healthy school snacks in municipal community centers and public health care centers | Principal of municipal community center, Nutritionist of community health center | Parents | Creating supportive environments | Municipal community center | 2 times |
| 20 | Appoint a task force to check the facilities around the schools | Physical education teacher, Parents | --- | Creating supportive environments | Local community | From the beginning of the intervention |
| 21 | Appoint a task force to record the programs implementation process (Fidelity assessment) | Principal of municipal community center, Nutritionist of the community health center, Parents | --- | Creating supportive environments | School, Municipal community center, Community health center | From the beginning of the intervention |
| **The average fidelity of implementation was 72.5 %**  **The average quality of implementation was 3.5** | | | | | | |
| **2. Promoting community knowledge of childhood obesity prevention efforts in target local communities** | | | | | | |
| 1 | Creating a separate school health channel in Telegram or WhatsApp | School consultant in health & education | Parents, School personnel | Education, Informing | School | From the beginning of the intervention |
| 2 | Enhance the schools’ and municipal community centers’ channel in Telegram or WhatsApp | School principal, School consultant in Health & education, Parents, Principal of municipal community center | Parents, School personnel | Education, Informing | -- | From the beginning of the intervention |
| 3 | Establishment of small informing group among parents | School principal, School consultant in health & education, Parents | Parents | Informing | School | From the beginning of the intervention |
| 4 | Informing about municipal community centers’ and public health care centers’ free services | Principal of municipal community center, Nutritionist of community health center | Parents, School personnel | Informing | School, Municipal community center, Public health care center | From the beginning of the intervention |
| **The average fidelity of implementation was 81 %**  **The average quality of implementation was 3.5** | | | | | | |
| **3. Promoting the local communities leaders’ support of childhood obesity prevention efforts** | | | | | | |
| 1 | In-person meetings with the principals of schools and municipal community centers and Nutritionists of community health center | School consultant in health & education, Research team member | School principals, Principles and/or nutritionists of the public health care centers and municipal community centers | Sensitizing | School, Municipal community center, Community health center | From the beginning of the intervention |
| 2 | Holding group discussions with representative parents of each school | School principal, School consultant in health & education, Research team member | Parents, School principals | Education, Sensitizing | School | From the beginning of the intervention |
| 3 | In-person lecture for members of the Parents-Teachers Association | School consultant in health & education, Research team member | Parents, School principals | Sensitizing | School | From the beginning of the intervention |
| 4 | In-person meetings with the school’s health ambassadors | School consultant in health & education, Research team member | Students, School principals | Education, Sensitizing | School | From the beginning of the intervention |
| **The average fidelity of implementation was 95 %**  **The average quality of implementation was 3.95** | | | | | | |
| **4. Promoting community climate/partnership to address childhood obesity in target local communities** | | | | | | |
| 1 | Holding educational program for parents in partnership with community health care center | School principal, School consultant in health & education, Nutritionist of community health center | Parents | Education, Sensitizing | School | 2 times |
| 2 | Holding face-to-face educational sessions for parents | Principal of municipal community center, Nutritionist of community health center | Parents | Education, Sensitizing | Municipal community center | Monthly |
| 3 | Holding face-to-face educational sessions with the topic of healthy foods and physical activity for students | School consultant in health & education, Nutritionist of community health center | Students | Education, Sensitizing | School | Monthly |
| 4 | Establishment of small informing group among parents | School principal, School consultant in health & education, Parents | Parents | Informing | School | From the beginning of the intervention |
| 5 | Establishment of small informing group among students | School consultant in health & education, Physical education teacher | Students | Informing | School | From the beginning of the intervention |
| 6 | Developing health massages in the schools | School consultant in health & education, Nutritionist of the community health center, Principal of municipal community center | Students, School personnel, Parents | Education, Creating supportive environments, Sensitizing | School, Municipal community center | From the beginning of the intervention |
| 7 | Encouragement of the students | School consultant in health & education, Physical education teacher | Students | Encouragement | School | From the beginning of the intervention |
| 8 | Merging several programs to direct the school and parental time | School principal, Parents | Parents, School personnel | Creating supportive environments | School | From the beginning of the intervention |
| **The average fidelity of implementation was 98 %**  **The average quality of implementation was 3.5** | | | | | | |
| **5. Promoting the local communities’ knowledge about childhood obesity** | | | | | | |
| 1 | Creating a separate school health channel in Telegram or WhatsApp | School consultant in health & education | Parents, School personnel | Education, Informing | School | From the beginning of the intervention |
| 2 | Establishment of small informing group among students | School consultant in health & education, Physical education teacher | Students | Informing | School | From the beginning of the intervention |
| 3 | Holding group discussions with teachers with the participation of the community health center | School principal, School consultant in health & education, Nutritionist of community health center | Teachers | Education, Sensitizing | School | 2 times |
| 4 | Holding face-to-face educational sessions with the topic of healthy foods and physical activity for students | School consultant in health & education, Nutritionist of community health center | Students | Education, Sensitizing | School | 2 times |
| 5 | Providing educational books/booklet for the library of school and classroom | School consultant in Health & education, Parents, Nutritionist of community health center | Students, Teachers | Creating supportive environments | School | From the beginning of the intervention |
| 6 | Providing pamphlets, essays, and educational content in schools’ and municipal community centers’ channels in Telegram or WhatsApp for parents | Principal of municipal community center, School consultant in health & education | Students, Parents, School personnel | Education, Creating supportive environments, Sensitizing | -- | From the beginning of the intervention |
| 7 | Holding educational program for parents in partnership with community health center | School principal, School consultant in health & education, Nutritionist of community health center | Parents | Education, Sensitizing | School | 2 times |
| 8 | Providing pamphlets, essays, and educational content by student health ambassadors | School consultant in health & education | Students | Education, Creating supportive environments, Encouragement | School | From the beginning of the intervention |
| 9 | Holding face-to-face educational sessions for parents | School consultant in health & education, Nutritionist of community health center | Parents | Education, Sensitizing | Municipal community center | Monthly |
| **The average fidelity of implementation was 78 %**  **The average quality of implementation was 3.4** | | | | | | |
| **6. Promoting available resources to address childhood obesity in target local communities** | | | | | | |
| 1 | In-person meetings with the principals of schools and municipal community centers | School consultant in health & education, Research team member | School principals, Principles and/or nutritionists of the public health care centers and municipal community centers | Sensitizing | School, Municipal community center | From the beginning of the intervention |
| 2 | Creating a separate school health channel in Telegram or WhatsApp | School consultant in health & education | Parents, School personnel | Sensitizing, Informing | -- | From the beginning of the intervention |
| 3 | Assessing around the school for organizing school physical activity classes and competitions | Parents | --- | Creating supportive environments | Local community | From the beginning of the intervention |
| 4 | Informing about municipal community centers’ and public health centers’ free services | Principal of municipal community center, Nutritionist of community health center | Parents, School personnel | Sensitizing, Informing | School, Municipal community center, Community health center | From the beginning of the intervention |
| **The average fidelity of implementation was 65 %**  **The average quality of implementation was 3.4** | | | | | | |

*Food & Nutrition Committee
