# Supplementary material for: Improving community readiness among Iranian local communities to prevent childhood obesity
Source: BMC Public Health. 2023 Feb 15;23:344. doi: 10.1186/s12889-023-15163-3 (PMC9931445; doi:10.1186/s12889-023-15163-3)
Supplement: Supplementary file 3 — Additional file 3. Data collecting form for assessing the quantitative and qualitative aspects of activities. [file 12889_2023_15163_MOESM3_ESM.docx]

Additional file **3:** Data collecting form for assessing the quantitative and qualitative aspects of activities

**Local community, No.**

**School name: …..**

**District: ……**

| **No.** | **Activities** | **^¶^Compliance** | **Date** | **Scale** | **Resources** | **Duration** | **Participants** | **Executive director** | ***Quality (1-5)** |
| --- | --- | --- | --- | --- | --- | --- | --- | --- | --- |
| 1 |  |  |  |  |  |  |  |  |  |
| 2 |  |  |  |  |  |  |  |  |  |
| 3 |  |  |  |  |  |  |  |  |  |
| 4 |  |  |  |  |  |  |  |  |  |
| 5 |  |  |  |  |  |  |  |  |  |
| 6 |  |  |  |  |  |  |  |  |  |
| 7 |  |  |  |  |  |  |  |  |  |

^¶^Yes/No; *Very low to very high

**Note:** ……………………………………………………………………………………………………………………………............…………………………………………………………………………………………………………………………………………………………………
