# Supplementary material for: Improving community readiness among Iranian local communities to prevent childhood obesity
Source: BMC Public Health. 2023 Feb 15;23:344. doi: 10.1186/s12889-023-15163-3 (PMC9931445; doi:10.1186/s12889-023-15163-3)
Supplement: Supplementary file 4 — Additional file 4. Interview consent form. [file 12889_2023_15163_MOESM4_ESM.docx]

Additional file **4:** Interview consent form


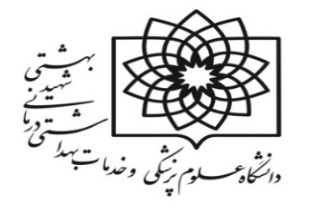


**Shahid Beheshti University of Medical Sciences**

**Name of Department:** Department of Community Nutrition, Faculty of Nutrition Sciences and Food Technology.

**Project title:** Community Readiness Improvement for Tackling Childhood Obesity (CRITCO): a community-based intervention program in Tehran.

**Description of the study:** Childhood obesity is currently one of the international public health concerns. According to the worrying upward trend of childhood obesity in Iran, interventions to improve children’s obesity by focusing on prevention efforts are essential. A readiness assessment is essential to inform planners about the feasibility of implementing a prevention program.

**Purpose of the interview:** The interview helps us determine the readiness stage of target communities to engage with childhood obesity prevention initiatives.

I confirm that I have read and understood the information about the project.

I confirm that I have had the opportunity to ask questions, and the researcher has answered any questions about the study to my satisfaction.

I understand that my participation is voluntary and that I am free to withdraw from the project at any time without giving a reason or consequences.

I understand that I can withdraw my data from the study at any time.

I understand that any information recorded in the investigation remains confidential, and no information identifying me is publicly available.

I consent to audio/ video/ interviews being recorded as part of the project.

**Name of participant: Date/Signature:**

**Name of interviewer: Date/ Signature**:
