# Supplementary material for: Improving community readiness among Iranian local communities to prevent childhood obesity
Source: BMC Public Health. 2023 Feb 15;23:344. doi: 10.1186/s12889-023-15163-3 (PMC9931445; doi:10.1186/s12889-023-15163-3)
Supplement: Supplementary file 5 — Additional file 5. The results of fidelity assessment by sex and aSES. [file 12889_2023_15163_MOESM5_ESM.docx]

Additional file **5:** The results of fidelity assessment by sex and **^a^**SES

| **Monitoring process** | **District 2 (High SES)** | | **District 16 (Low SES)** | |
| --- | --- | --- | --- | --- |
|  | Girls’ school | Boys’ school | Girls’ school | Boys’ school |
| Compliance with the intervention activities (%) | 81 | 77 | 89 | 79 |
| Quality of implemented activities | 3.75 | 3.2 | 3.9 | 3.25 |

^a^SES; socio-economic status
